# Dental caries and periodontal disease among people who use drugs: a systematic review and meta-analysis

**DOI:** 10.1186/s12903-020-1010-3

**Published:** 2020-02-10

**Authors:** Mohsen Yazdanian, Bahram Armoon, Alireza Noroozi, Rasool Mohammadi, Amir-Hosein Bayat, Elahe Ahounbar, Peter Higgs, Hormoz Sanaei Nasab, Azadeh Bayani, Morteza Hemmat

**Affiliations:** 1 Research Center for Prevention of Oral and Dental Diseases, Baqiyatallah University of Medical Sciences, Tehran, IR Iran; 2 Iranian National Center for Addiction Studies, Tehran University of Medical Sciences, Tehran, Iran; 3 Department of Public Health,, Loresatn University of Medical Sciences, Khoram Abad, Iran; 4 Social Determinants of Health Research Center, Saveh University of Medical Sciences, Saveh, Iran; 5 Department of Public Health, School of Psychology and Public Health, La Trobe University, Melbourne, Australia; 6 Health Research Center, Life Style Institute, Baqiyatallah University of Medical Sciences, Tehran, Iran; 7 Student Research Committee, School of Allied Medical Sciences, Shahid Beheshti University of Medical Sciences, Tehran, Iran

**Keywords:** Oral health index, Periodontal disease, DMFT, Drug use

## Abstract

**Background:**

The aim of our study was to perform a systematic review of the literature and meta-analysis in order to investigate relationship between drug use and oral health.

**Methods:**

We searched for studies in English published before July 1, 2019 on PsycINFO, PubMed, SciELO, Scopus, and Web of Science. We assessed the relationship between drug use (methamphetamines, heroin; opiates; crack, cocaine and cannabis as dependent variables) and reported tooth loss, periodontal disease, or decayed, missing, and filled teeth index as an independent variable. The data were analyzed using Stata 12.0 software.

**Results:**

We initially identified 1836 potential articles (with 1100 duplicates) and screened the remaining 736 titles and abstracts, comprising 54 studies. In the next step, we evaluated the full-texts; 44 studies were excluded, accordingly. In total, we included 10 publications in the meta-analysis. Drug type was associated with periodontal disease (OR 1.44; 95% CI 0.8–2.6) and pooled estimates showed that type of drug used increased the odds of the number of decayed, missed and filled teeth (DMFT) (OR 4.11; 95% CI 2.07–8.15) respectively.

**Conclusions:**

The analytical challenges of segregating the impact of individual drug types on oral health diseases mean that investigations on the direct relationship between oral health status and drug use are limited. Developing programs to improve potential confounding with various substances and addressing the dental health needs of people who use drugs is vital if we are to improve their overall quality of life.

## Background

Problematic and dependent drug use is associated with economic and social problems and is often associated with a range of medical complications [1]. The most commonly used illicit drugs globally are cannabis, opioids and stimulants [2]. The criminalization of drug use has serious public health consequences that adversely impact on the global community [3–5]. Previous studies have highlighted the negative health consequences of some drugs for people who use them [6–8]. In relation to oral health specifically, there is evidence that people who use drugs (PWUD) have a high sugar intake [9, 10]. Several studies indicate that exposing to long term drug usage are more susceptible to high intake of sugar [11, 12]. According to these studies individuals who had chronic intake of drugs such as opioid showed higher intake of sugary food and less complex carbohydrates, fruits, vegetables and fats from fish [9, 13] and do not seek help when symptoms of swelling and pain appear in their mouth [14]. Different drugs can affect the soft and hard tissues of the mouth may result in malignant states or could predispose people to oral infections [15]. Caries are one of the most serious diseases of the mouth and can be prevented using prophylactic and protective methods [16]. The caries risk factors studied in clinics and the medical practitioner evaluates signs and symptoms of salivary hypofunction, dietary practices and measures of oral hygiene [17].

Saliva and its components are considered as effective protective endogenous agents [16], with hyposecretion of saliva by salivary glands being one of the most critical factors that increase the risk of caries [18]. Furthermore, there are the higher prevalence of periodontal diseases in methamphetamine (MA) users and some variables such as xerostomia, high carbohydrate diet, and poor oral hygiene [19, 20], endocrine dysfunction and decreased body immunity [21] are involved in poor oral and dental disease in the users. Also, several lines of evidence showed pathologic damages in oral hard and soft tissues among PWUD and these pathological changes correlate to the higher prevalence of caries with the certain substances [22–25]. Some studies indicated that drugs such as MA have a negative effects on oral health [26, 27], and two studies reported that there was no impact [17, 28]. It seems MA use causes destructive dental caries. Some studies have suggested that it may be the physical or chemical qualities of MA or its components, such as their toxicity or acidity, directly attacking physical structure of tooth [29]. Other investigations showed that MA leads to dry mouth which decreases protective functions of saliva and mucus [26, 30, 31]. Furthermore, dental caries might happen secondary to prolonged drug-use and associated health behaviors has been attributed to salivary malfunction, poor oral hygiene, and consumption of refined carbohydrates [32]. According to a systematic review of MA use and health outcomes among adolescents, there is insufficient evidence of an association between dental outcomes and MA use [33]. Nonetheless, another study showed higher rates of dental disease among adult people who use MA [34]. In fact, dental disease as one of the most important comorbidities in PWUD is a reasons to develop treatment plans that address both oral health problems and drug use [34]. Also, one applicable index for assessing the condition of dental and oral health is the number of decayed, missed and filled teeth (DMFT) and this index comprises the number of decayed, missing, and filled teeth in a person [35]. Moreover, studies investigating oral health status have reported DMFT as a scientifically accepted index to elucidate the condition of dental and oral health [16, 18, 22, 36–39]. Thus, the aim of our study was to perform a systematic review of the literature and meta-analysis in order to investigate relationship between drug use and oral health condition.

## Methods

We followed the items that were preferred for Systematic Reviews and Meta-Analyses (PRISMA) guideline for conducting the current systematic review study [40].

### The questions of the systematic review

We considered the below questions for this aim:

Is there an association between drug type and oral health conditions (DMFT and periodontal disease) among PWUD?

Considering relevant MeSH terms on the basis of PICO model and in accordance with the questions specified in advance, we conducted our search strategy, as follows:

(a) P: Methamphetamine Users; heroin users; opiate users; crack users; (b) I: people who use drugs; (c) C: people who do not use drugs; (d) O: DMFT/periodontal disease.

The detailed search for each specific database can be found in Table 1.

**Table 1:** search strategy

Database	Key words
PsycINFO	(“Drug abuse” OR “drug use OR inject drug use OR Methamphetamine user OR Cannabis users OR Heroin users OR Marijuana users OR Opiate users OR Amphetamines users or Cocaine users or Hallucinogens users”) AND (“Dental Caries” OR “Periodontal Diseases” OR “Periodontitis” OR “DMF Index” OR “Tooth Loss” OR “Edentulism” OR “Dental Status” OR “Oral Health”)
Scielo	Drug abuse [Title words] or drug use [Title words] or inject drug use [Title words] or Methamphetamine users [Title words] or Cannabis users [Title words] or Heroin users [Title words] or Marijuana users [Title words] or Opiate users [Title words] or Amphetamines users [Title words] or Cocaine users [Title words] or Hallucinogens users [Title words]and Oral health [Title words] or Dental Caries [Title words] or periodontal [Title words] or DMF Index [Title words] or oral hygiene [Title words] or decayed, missed and filled teeth [Title words] or tooth Loss [Title words] or “Edentulism” [Title words] or “Dental Status” [Title words] or “Oral Health” [Title words]
Pubmed	#22 Search (((((((((((Substance-Related Disorders[MeSH Terms]) OR Substance Abuse, Intravenous[MeSH Terms]) OR drug use[Title]) OR inject drug use[Title]) OR Methamphetamine user[Title]) OR Cannabis users[Title]) OR Heroin users[Title]) OR Marijuana users[Title]) OR Opiate users[Title]) OR Cocaine [Title]) Hallucinogen [Title]) OR Amphetamines users[Title])) AND (((((((((Dental Caries[MeSH Terms]) OR Periodontal Diseases[MeSH Terms]) OR Periodontitis[MeSH Terms]) OR DMF Index[MeSH Terms]) OR Tooth Loss[MeSH Terms]) OR Edentulism[Title]) OR Dental Status[Title]) OR Oral Health[MeSH Terms]) OR Oral Hygiene Index[MeSH Terms]) #21 Search ((((((((Dental Caries[MeSH Terms]) OR Periodontal Diseases[MeSH Terms]) OR Periodontitis[MeSH Terms]) OR DMF Index[MeSH Terms]) OR Tooth Loss[MeSH Terms]) OR Edentulism[Title]) OR Dental Status[Title]) OR Oral Health[MeSH Terms]) OR Oral Hygiene Index[MeSH Terms] #20 Search (((((((((Substance-Related Disorders[MeSH Terms]) OR Substance Abuse, Intravenous[MeSH Terms]) OR drug use[Title]) OR inject drug use[Title]) OR Methamphetamine user[Title]) OR Cannabis users[Title]) OR Heroin users[Title]) OR Marijuana users[Title]) OR Opiate users[Title]) OR Amphetamines users[Title] #19 Search Oral Hygiene Index[MeSH Terms] #18 Search Oral Health[MeSH Terms] #17 Search Dental Status[Title] #16 Search Edentulism[Title] #15 Search Tooth Loss[MeSH Terms] #14 Search DMF Index[MeSH Terms] #13 Search Periodontitis[MeSH Terms] #12 Search Periodontal Diseases[MeSH Terms] #11 Search Dental Caries[MeSH Terms] #10 Search Amphetamines users[Title] #9 Search Opiate users[Title] #8 Search Marijuana users[Title] #7 Search Heroin users[Title] #6 Search Cannabis users[Title] #5 Search Methamphetamine user[Title] #4 Search inject drug use[Title] #3 Search drug use[Title] #2 Search Substance Abuse, Intravenous[MeSH Terms] #1 Search Substance-Related Disorders[MeSH Terms]
Scopus	(TITLE-ABS-KEY (drug AND abuse) OR TITLE-ABS-KEY (drug AND use) OR TITLE-ABS-KEY (inject AND drug AND use) OR TITLE-ABS-KEY (methamphetamine AND user) OR TITLE-ABS-KEY (cannabis AND users) OR TITLE-ABS-KEY (heroin AND users) OR TITLE-ABS-KEY (marijuana AND users) OR TITLE-ABS-KEY (opiate AND users) OR TITLE-ABS-KEY (amphetamines AND users) AND TITLE-ABS-KEY (dental AND caries) OR TITLE-ABS-KEY (periodontal AND diseases) OR TITLE-ABS-KEY (periodontitis) OR TITLE-ABS-KEY (dmf AND index) OR TITLE-ABS-KEY (tooth AND loss) OR TITLE-ABS-KEY (edentulism) OR TITLE-ABS-KEY (dental AND status) OR TITLE-ABS-KEY (oral AND health))
Web of Knowledge	TS = (Substance-Related Disorders OR Substance Abuse, Intravenous OR drug use OR inject drug use OR Methamphetamine user OR Cannabis users OR Heroin users OR Marijuana users OR Opiate users OR Amphetamines users) AND TS = (Dental Caries OR Periodontal Diseases OR Periodontitis OR DMF Index OR Tooth Loss OR Edentulism OR Dental Status OR Oral Health OR Oral Hygiene Index)
Cochrane	#1 MeSH descriptor: [Substance-Related Disorders] explode all trees #2 MeSH descriptor: [Substance Abuse, Intravenous] explode all trees #3 (“drug user”):ti,ab,kw #4 (inject drug user):ti,ab,kw #5 (Methamphetamine user):ti,ab,kw #6 (Cannabis users):ti,ab,kw #7 (Heroin users):ti,ab,kw #8 (Marijuana users):ti,ab,kw #9 (Opiate users):ti,ab,kw #10 (Amphetamines users):ti,ab,kw #11 #1 OR #2 OR #3 OR #4 OR #5 OR #6 OR #7 OR #8 OR #9 OR #10 #12 MeSH descriptor: [Dental Caries] explode all trees #13 MeSH descriptor: [Periodontal Diseases] explode all trees #14 MeSH descriptor: [Periodontitis] in all MeSH products #15 MeSH descriptor: [DMF Index] explode all trees #16 MeSH descriptor: [Tooth Loss] explode all trees #17 (“edentulism”):ti,ab,kw #18 (Dental Status):ti,ab,kw #19 MeSH descriptor: [Oral Health] explode all trees #20 MeSH descriptor: [Oral Hygiene Index] explode all trees #21 #12 OR #13 OR #14 OR #15 OR #16 OR #17 OR #18 OR #19 OR #20 #22 #11 AND #21

#### Inclusion and exclusion criteria

Original cross-sectional and longitudinal prospective and retrospective observational studies were included. Studies compared PWUD with people who do not use drugs (PWDNUD) in terms of oral health conditions (DMFT/periodontal disease). The representativeness of the sample as well as adequacy of power for determining statistical significance could be ensured by either clarifying the representativeness of sample (e.g., the representativeness of the sub-sample of a national study) or providing sample selection details. Studies of other specific samples, such as psychiatric populations, and people who use alcohol or smoke tobacco were excluded from the study. We excluded qualitative research studies, in vitro investigations, animal studies, reviews, case reports and series, letters to editor, and congress abstracts.

#### Outcome measure

Studies that reported the effect of using a specific type of drug use on oral health conditions (DMFT/periodontal disease).

#### Definitions/criteria considered for PWUD

We included articles on illicit drug use (i.e. methamphetamines, heroin, opiates, cocaine, cannabis and crack) based-on self-report measures or interviews.

#### Definitions/criteria considered for oral health conditions

We only included oral diseases considered as public health issues with a global burden. As a result, investigations reporting tooth loss, periodontal disease, or DMFT as an outcome were included in the review.

We evaluated this by two most frequently used indexes in the epidemiological researches which consisted of the DMFT: the number of decayed, missing and filled teeth and the DMFS: the number of decayed, missing and filled surfaces. In the DMFT score the unit for measuring is every tooth, while for the DMFS is dental surface. Frontal teeth have four surfaces, while the back teeth have five. Hence the maximum DMFT achieves 32 (but we often do not consider wisdom teeth, considering the maximum 28), while the maximum DMFS is 148 (or 128 if we don’t consider wisdom teeth) [41].

Periodontal disease is defined the chronic inflammation of the supporting structures of the teeth. It occurs as gingivitis which is reversible demolishing related to the gingiva and may cause to the periodontitis. Irreversible demolishing of the gingiva, bone and periodontal ligaments that hold teeth in place. The stage of it is computed by a manual probe to evaluate pocket probing depth (PPD) or clinical attachment level (CAL) [42]. While the threshold of PPD more than 3 mm or CAL of more than 2 mm are for periodontitis [43], other researchers indicate that the threshold for PPD should be considered 4 mm [44]. Generally, if more than 4–5 mm of bone around a tooth is lost, the tooth will be increasingly movable until it falls out. In the evaluation, a PPD of 4–5 mm is considered as a ‘shallow’ pocket, although ‘deep’ pockets are 6 mm or higher [45].

All studies included clinical measures or participant self-reports showing the presence of oral diseases. It is worth noting that we selected the most severe oral health condition in the presence of several oral disease categories. Edentulism and tooth loss were assessed individually. Moreover, we excluded investigations on dysfunction temporomandibular, erosion, or xerostomia. As noted above all qualitative studies focusing on dental outcomes, including poor oral health status were excluded.

#### Search strategy and study selection

We searched studies in English languages published before July 1, 2019 on PsycINFO, PubMed, SciELO, Scopus, and Web of Science. Table 1 describes search strategies in terms of each database. The surveyed references were managed in EndNote X7 software (Thomson Reuters, New York, NY, USA). We excluded duplicate identified studies. Two independent reviewers (AB and BA) investigated the titles and abstracts, in accordance with the inclusion and exclusion criteria of the study. Any disagreements were discussed by the two reviewers until consensus reached. A third (AMB) person from research team provided input as needed. Then, these reviewers reviewed the full-texts, observing the inclusion and exclusion criteria. Apart from the aforementioned electric search, a manual search of the reference lists was conducted on all the included studies.

#### Data extraction and quality assessment

Data items were extracted from each selected study included the first author’s name and publication year, sample characteristics, location, and design of the study, as well as data on the exposure and outcomes variables. In addition, we recorded confounding factors, effect measure, performed adjustments, and statistical methods. We contacted the relevant authors and made necessary clarifications, if necessary. The reviewers independently employed previously defined worksheets for obtaining the required data. In the first step, the reviewers observed and omitted the duplicated title and abstract which had 89% agreement according to the criteria one through three explained below. In the second step titles/abstracts met these defined criteria were selected to full-text review according to the inclusion criteria (96% agreement). For the quality assessment, we applied the unweighted kappa to evaluate the agreement between the two authors (BA and AB). We represented the levels of agreement including poor, slight, fair, moderate, substantial and complete by the values of 0, 01–0.02, 0.021–0.04, 0.041–0.06, 0.061–0.08, and 0.081–1.00, respectively [46].

#### Assessment of risk of Bias in included studies

We surveyed the included studies with respect to the quality of their methodology applying the Critical Appraisal Checklist for observational studies by The Joanna Briggs Institute (JBI) [47] . There are 10-item tool consisting of “Yes,” “No,” or “Unclear” options for cohort, case-control studies, and 8-items for cross-sectional studies answered by reviewers. The total score of each study equals to the total number of ‘yes’ answers, ranging 0–10. We classified the publications as: low quality (0–3 scores); medium quality (4–6 scores); and high quality (7–10 scores). The same reviewers independently conducted the data extractions and quality survey; any disagreements were solved through discussion (Tables 2, 3 and 4).

**Table 2:** Risk of bias assessment using JBI’s critical appraisal tools for cross sectional studies

Items for critical appraisal	Were the criteria for inclusion in the sample clearly defined?				Were the study subjects and the setting described in detail?				Was the exposure measured in a valid and reliable way?				Were objective, standard criteria used for measurement of the condition?				Were confounding factors identified?				Were strategies to deal with confounding factors stated?				Were the outcomes measured in a valid and reliable way?				Was appropriate statistical analysis used?				Overall appraisal	Quality of the evidence
studies	yes	No	Unclear	Not applicable	yes	No	Unclear	Not applicable	Yes	No	Unclear	Not applicable	Yes	No	Unclear	Not applicable	Yes	No	Unclear	Not applicable	Yes	No	Unclear	Not applicable	Yes	No	Unclear	Not applicable	Yes	No	Unclear	Not applicable		
Rooban et al. [48]	√				√					√			√				√					√			√				√				Included	moderate
Reece [49]	√				√				√				√					√			√				√					√			Included	High
Kayal et al. [50]	√				√				√				√					√				√			√					√			Included	moderate
D’Amore et al. [51]	√				√				√				√				√					√			√					√				High
Gupta et al. [52]	√				√				√				√					√					√				√			√				moderate

**Table 3:** Risk of bias assessment using JBI’s critical appraisal tools for cohort studies

Items for critical appraisal	Were the two groups similar and recruited from the same population?				Were the exposures measured similarly to assign people to both exposed and unexposed groups?				Was the exposure measured in a valid and reliable way?				Were confounding factors identified?				Were strategies to deal with confounding factors stated?				Were the groups/participants free of the outcome at the start of the study (or at the moment of exposure)?				Were the outcomes measured in a valid and reliable way?				Was the follow up time reported and sufficient to be long enough for outcomes to occur?				Was follow up complete, and if not, were the reasons to loss to follow up described and explored?				Were strategies to address incomplete follow up utilized?								Overall appraisal	Quality of the evidence
studies	yes	No	Unclear	Not applicable	yes	No	Unclear	Not applicable	Yes	No	Unclear	Not applicable	Yes	No	Unclear	Not applicable	Yes	No	Unclear	Not applicable	Yes	No	Unclear	Not applicable	Yes	No	Unclear	Not applicable	Yes	No	Unclear	Not applicable	Yes	No	Unclear	Not applicable	Yes	No	Unclear	Not applicable	Yes	No	Unclear	Not applicable		
Thomson et al. [53].	√				√					√			√				√							√	√						√		√				√				√				Included	High

**Table 4:** Risk of bias assessment using JBI’s critical appraisal tools for case-control studies

Items for critical appraisal	Were the groups comparable other than the presence of disease in cases or the absence of disease in controls?				Were cases and controls matched appropriately?				Were the same criteria used for identification of cases and controls?				Was exposure measured in a standard, valid and reliable way?				Was exposure measured in the same way for cases and controls?				Were confounding factors identified?				Were strategies to deal with confounding factors stated?				Were outcomes assessed in a standard, valid and reliable way for cases and controls?				Was the exposure period of interest long enough to be meaningful?				Was appropriate statistical analysis used?								Overall appraisal	Quality of the evidence
studies	yes	No	Unclear	Not applicable	yes	No	Unclear	Not applicable	Yes	No	Unclear	Not applicable	Yes	No	Unclear	Not applicable	Yes	No	Unclear	Not applicable	Yes	No	Unclear	Not applicable	Yes	No	Unclear	Not applicable	Yes	No	Unclear	Not applicable	Yes	No	Unclear	Not applicable	Yes	No	Unclear	Not applicable	Yes	No	Unclear	Not applicable		
Morio et al. [27]	√				√					√			√				√					√			√				√				√				√				√				Included	High
Shetty et al. [54]	√				√				√											√	√				√					√			√				√				√				Included	High
Rommel et al. [55]			√		√				√				√						√		√				√				√				√				√				√				Included	High
Nives Protrka et al. [24]		√					√		√				√				√				√				√						√		√				√				√				included	moderate

#### Statistical analysis

An individual meta-analysis was conducted for each oral disease. An independent analysis was also performed on studies where more than 2 variables of interest were presented. The adjusted data were considered in the meta-analysis. In other cases, we considered or calculated crude result estimates. The present study applied Odds Ratio (OR) for measuring the effect size with 95% confidence interval (CI). We converted the studies’ relative risk measures to ORs [56]. In order to calculate pooled ORs, we used fixed- and random-effects models. In addition, random-effects model was selected in case heterogeneity was observed [57]. Using I^2^ statistic (I^2^ of greater than 50%), heterogeneity was measured. The effect of each study on the pooled data were observed using sensitivity analysis. Eventually, the obtained data were analyzed in Stata 12.0 software (Stata Corp, College Station, TX, USA) and R 3.5.1 with the “meta” package was applied to conduct the meta-analysis.

## Results

### Study selection

**Fig. 1:**
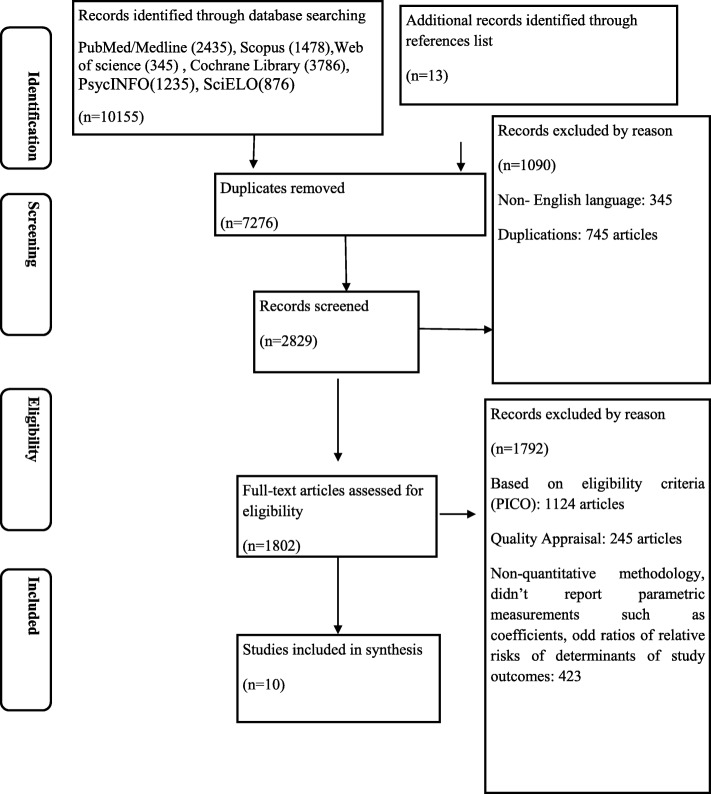
PRISMA flow diagram

Initial screening identified 1836 potential articles (with 1100 duplicates) by the electronic database searches. We then screened 736 titles and abstracts, with 54 studies being included. In the next step, we evaluated the full-texts of the manuscripts and 44 studies were excluded. In total, we included 10 publications in the final meta-analysis. Figure 1 represents PRISMA statement-based inclusion criteria of the study.

### Study characteristics

Table 5 list the most important aspects of the included studies in the meta-analysis, with respect to the correlations between publications.

**Table 5:** Main characteristics of the studies selected for the systematic review and meta-analysis with oral health as exposure 2019

author	Drug used by participants	Sample size	year	country	design	Quality of the evidence
Morio et al. [27]	Methamphetamine users	18	2008	USA	Case-control	High
Shetty et al. [54]	Methamphetamine users	571	2016	USA	Case-control	High
Rommel et al. [55]	Methamphetamine users	200	2016	Germany	Case-control	High
Thomson et al. [53].	Cannabis users	1015	2008	New Zealand	Cohort	High
Nives Protrka et al. [24]	Heroin users	200	2013	Croatia	Case-control	Moderate
Rooban et al. [48]	Heroin and Cannabis users	100	2008	India	Cross-section	Moderate
Reece [49]	Opiate users	233	2007	Australia	Cross-section	High
Kayal et al. [50]	Amphetamines	57	2014	Saudi Arabia	Cross-section	Moderate
D’Amore et al. [51]	Opioid and Marijuana users	563	2011	USA	Cross-section	High
Gupta et al. [52]	illicit drug users	126	2012	*India*	Cross-section	Moderate

### Synthesis of results/meta-analysis

#### Periodontal disease compared to drug type used

**Fig. 2:**
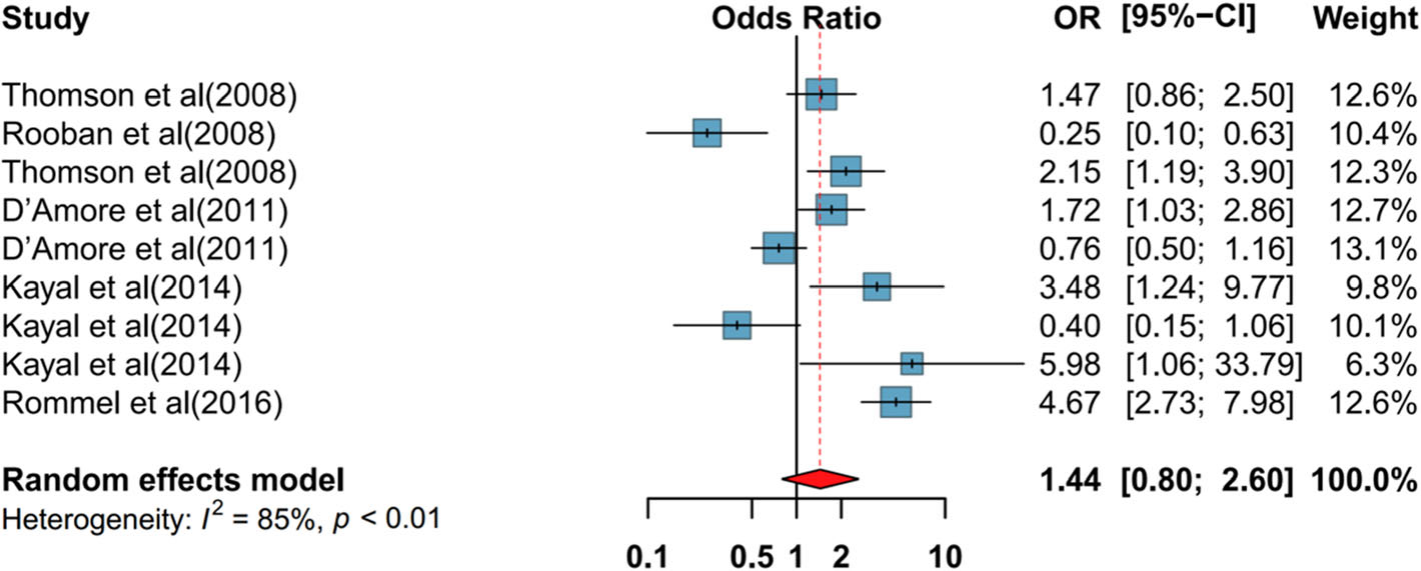
Pooled effect of type of drug use on periodontal status. CI confidence interval

**Fig. 3:**
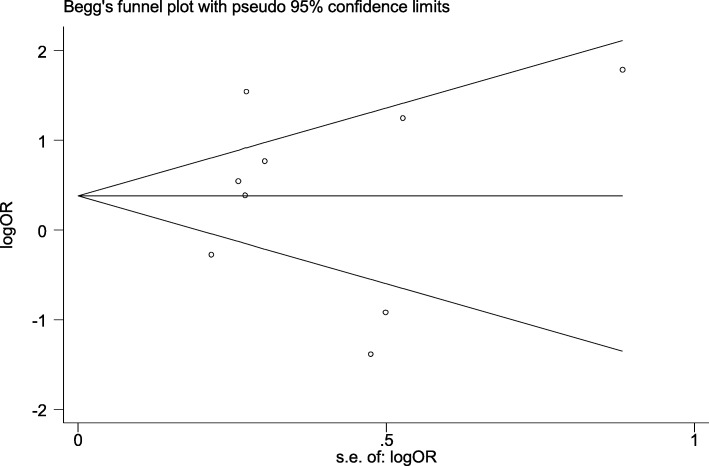
Begg’s funnel plot for assessing publication bias for studies that periodontal status reported

In our meta-analysis we considered 5 studies evaluating the relationship between drug type and periodontal disease [48, 50, 51, 53, 55]. These studies described the relationship between drug type as an exposure variable and periodontal disease as an outcome variable. These studies were conducted between 2008 to 2016, the sample sizes were from 57 to 1015 with a high quality structured approach. Four studies were implemented in high-income country (such as USA, Germany, New Zealand, Saudi Arabia) [50, 51, 53, 55], and the study of Rooban et al. [48] was completed in India. The three of considered studies used cross-sectional analysis [48, 50, 51], one of them was a cohort study [53] and the last one used case-control design [55] and evaluated drug type using a self-report questionnaire. Regarding the oral health measure, the five studies applied bleeding on probing index (BOP) and periodontal screening index (PSI). Pursuant to pooled estimate, type of drug used was related with periodontal disease (OR 1.44; 95% CI 0.8–2.6) (Fig. 2). Five studies statistically monitored the analyses for potential confounders. Begg’s test found no publication bias (1.83, *P* = 0.62) or funnel plot analysis for periodontal status were existed. (Fig. 3).

#### DMFT index compare to drug type used

Seven studies [24, 27, 48, 49, 52, 54, 55] examined the relationship between the type of drug used and DMFT index among PWUD. Five studies were conducted in high-income countries [24, 27, 49, 54, 55], and two in a low middle-income country (India) [48, 52]. The date of studies ranged from 2007 to 2016, and the sample sizes were between 8 and 571. All had high quality approaches and three studies were categorized as having moderate quality of evidence. Four studies used a case-control approach for their analysis [24, 27, 54, 55] and three studies used cross–sectional design [48, 49, 52].

**Fig. 4:**
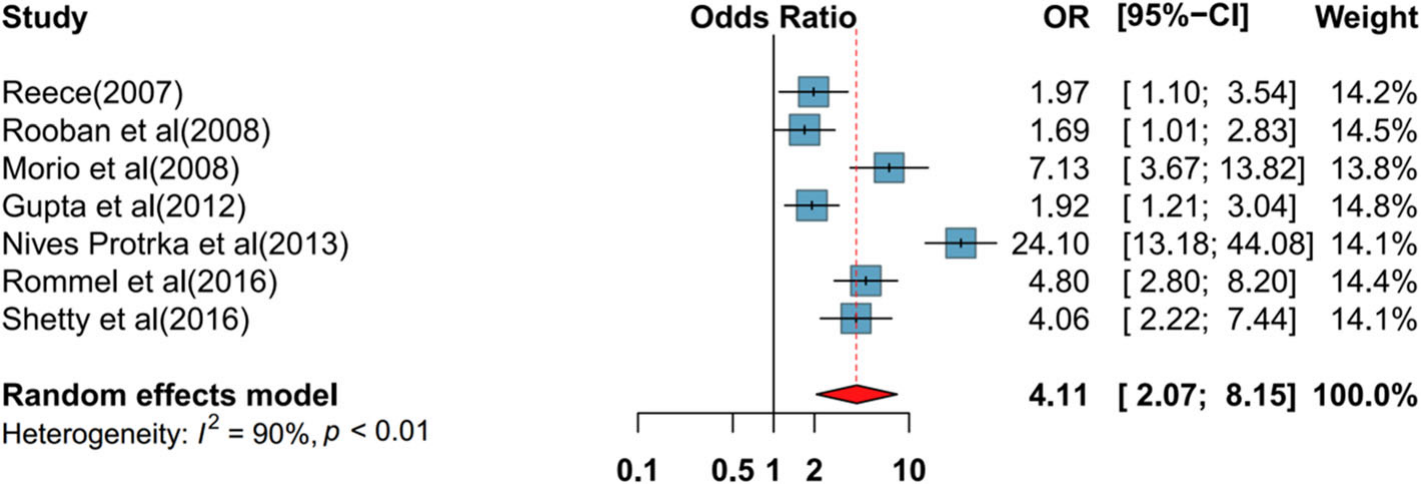
Pooled effect of type of drug use on DMFT. CI confidence interval

**Fig. 5:**
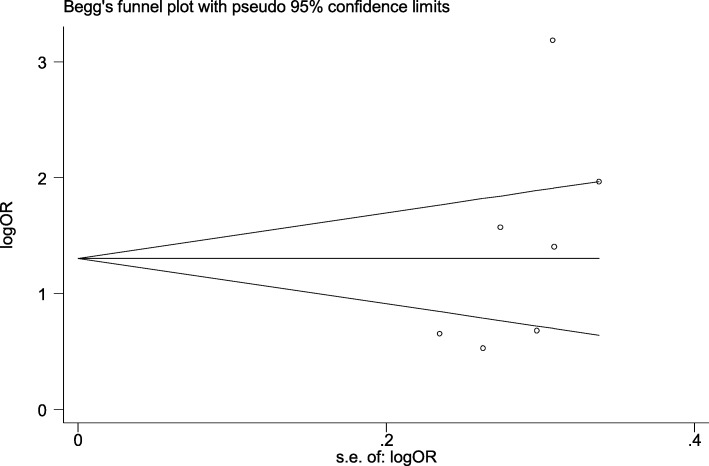
Begg’s funnel plot for assessing publication bias for studies that DMFT reported

The results demonstrated a positive relationship on the pooled estimate for type of drug used as an exposure variable for DMFT index. The specific type of drug of used showed 4.11 times higher odds of DMFT index (OR 4.11; 95% CI 2.07–8.15) (Fig. 4). For potential confounders all studies included in this study statistically monitored their analyses. In the final model there was heterogeneity of 90.5% among studies. There was no available body of facts presenting the publication bias in Begg’s funnel plot and Egger’s test (1.65, *P* = 0.11) (Fig. 5).

## Discussion

Prior empirically-based investigations and case studies have explored the relationship between oral health status and specific illicit drugs; however, our review of the literature resulted in no previous meta-analysis on this topic among PWUD. As a result, the current research assessed available empirical and clinical data with regard to the correlation between illicit drugs and significant oral conditions such as dental caries and periodontal disease among PWUD. Previous studies identified dental caries as the most prevalent condition among PWUD [19, 58]. Moreover, dental caries are more prevalent and severe among people who use MA, when compared to non-drug using controls [19, 55, 59]. According to a study among people who use MA in United States, untreated dental caries and the odds of having dental carries were respectively two and four times higher among cases, compared to the controls (i.e., National Health and Nutrition Examination Survey (NHANES) control group) [54]). In addition, the odds of reporting decayed, missing, or filled teeth were double among people who use MA, compared to the NHANES participants [29]. Consistent with the prior research, the mean scores of tooth decay (TD), missing teeth (MT) and DMFT were higher in the patients self-reporting drug use, in comparison to people who do not use drugs [19, 55, 59].

It could be assumed that people who use MA overlook their oral health status, given the significantly lower number of FT and higher DT scores in former MA users [60]. Another prevalent oral health problem among PWUD is periodontal disease. The corresponding reference data in the third Chinese National Epidemiological Sampling Survey of Oral Health was lower than the findings in respect of the frequency of periodontal pockets and deep periodontal pocket, and gingival bleeding in former users of crystal MA [61].

In addition, consistent with prior research, the obtained mean score of CPI was high in people who use MA [27]. The longer the duration of drug use, the greater the risk for oral health problems [62–64]. This is while the obtained scores of DT, DMFT and CPI were significantly greater in patients reporting ≥4 years of MA use, in comparison to individuals who reported shorter history of such use. This finding is in line with previous studies. In other words, the shorter the history of MA use, the better the status of oral health, including caries and periodontal diseases would be. There are major differences between the lifestyle of patients with drug use disorders and the general population. Overlooking oral health/hygiene status is prevalent among PWUD [19, 20, 55, 65–67]. Brushing teeth more than two times a day was significantly associated with lower TD score, compared to “regularly brushing” teeth [68]; this outcome highlights the importance of specific oral health education in people who use MA. Prior research suggests a strong association between poor oral hygiene and dental caries among people who report MAuse [19, 20]. Analyzing the individual components of the mean value of DMFT indicated the mean frequency of tooth decay shaped the significant part of index. This result reveals the necessity of dental treatment forPWUD. Also, the frequency of filled teeth was significantly lower in PWUD suggesting comprehensive dental care was rarely provided to this group. Such data show that PWUD not only suffer from poor oral health status and its associated complications, but they also fail to easily access affordable oral health care services. It is worth noting that the pharmacological effects of some drugs may mask the symptoms of caries and that PWUD may self-medicate in the face of severe pain [69].

Consistent with previous research, it was found that the risk of developing caries is significantly greater in patients with chronic MA use. However, few cases were identified suffering from “meth mouth syndrome” where the typical symptoms of rampant caries at labial and a proximal surfaces [70, 71]. Case reports that applied clinical assessments along with radiography provided more accurate results and may be the reason for the great frequency of carious lesions documented in comparison with these data. These findings are in line with the previous data suggesting a higher frequency of oral health problems among PWUD [17, 72, 73].

A previous study has documented perceived poor oral health perception and drug use as correlated [74, 75]. In addition, feelings of embarrassment and low self-esteem induced by unsatisfactory oral health were common among people using MA. The literature review identified perceived oral health as important in health-related quality of life [76, 77]. Considering the aforementioned findings, addressing the particular oral health concerns of people who use MA is important. Oral health services could improve the self-esteem of their MA patients in the form of the basic behavioral-based treatments alongside dental care.

The prevalence of periodontal disease was unexpectedly high among study participants. The prevalence of total periodontitis in the US general adult population aged 35–49 years is 37% [78]; however, more than 89% of the people who use MA reported total periodontitis. The severe periodontal disease risk indicators among MA users were consistent with the data obtained from the general US population; however, these studies differ in other dimensions. The severe periodontal disease risk was greater in older and African-American individuals in this cohort study people who use MA, which is expectable in the general US population. This is while smoking and education (a proxy for socioeconomic status) were not significantly correlated with severe periodontal disease in the MA cohort. However, these are considered as significant risk factors for the general US population. In addition, there was no significant correlation between the current status of smoking and severe periodontitis under the condition of controlling sociodemographic risk factors and MA use severity; however, the same variable had a relationship with root caries and untreated anterior dental caries. The reason for such associations remains unclear; concurrent cigarette smoking among people who use MA may implicate risk-promoting behaviors for dental caries. Some examples of such behaviors are smoking as the route of MA administration or sugar-sweetened beverages intake both which lead to generating tooth decay [79]. Considering the above-mentioned points, there is a high risk for generating moderate to severe periodontitis among PWUD; however, such correlation has been overlooked by scholars. Thomson et al. [53] investigated the relationship between periodontal diseases and cannabis smoking, and recognized cannabis use as an independent risk factor for developing periodontal diseases. The prevalence of poor oral health and severe periodontal diseases have been reported to be high among people who use heroin [79, 80]. It was also found that there is a strong correlation between greater attachment loss and heroin use in comparison with other drug types. Khocht et al., found no statistically significance relationship between attachment loss and cocaine use [81] but the difference might be explained by the small number of cocaine dependent individuals in the study. Prior research on MA has focused on the so called ‘meth mouth’ condition with limited attention to other periodontal conditions. The obtained data revealed increased attachment loss among people who use MA, compared to the general population; however, the correlation was not statistically significant. Some factors related to lifestyle such as poor nutrition, oral hygiene, and limited access to dental care may affect periodontal health status in PWUD [82–84]. According to prior research [80], the periodontal health status of patients with drug use disorders is poor. Such complication may be due to concomitant heavy use of tobacco and poor oral hygiene among them. In this regard, different substances (especially opiates) negatively effect cell division; as a result, they tilt the balance towards tissue breakdown and impair its repair and regeneration [49].

The limitations of our systematic review include the exclusion of studies on homeless populations and studies of other high-risk communities, such as people hospitalized for mental health problems or people who suffer from periodontal diseases. Moreover, we recommend assessing the studies considering the population who were mentioned above, because the high risk population may have different risk factors. Furthermore, most of the included studies were cross-sectional and this may restrict causal and temporal deduction on the relationship between oral diseases and drug use. This meta-analyses may enhance the statistical inference of analyses and are discussed as reliable sources of evidence. Another limitation is that a few studies investigated the association between dental caries and drug use, emphasizing this gap in the literature. Also, since we did not interfere with the setting of independent and dependent variables, we had to report only the data that were published in the articles. Although the associations of any systemic disorders, age and Psychological condition with increased prevalence of caries among PWUDs were necessary for suitable interventions but only one paper reported the association between the age and periodontal diseases, none of them reviewed the relationship between psychological condition and any systemic disorders with any systemic disorders and therefore we could not report it in our results because of the few numbers of them. The strengths of our study include the number of high quality studies reviewed with a large representative sample and multivariate analysis regulating for potential confounders. These factors provided greater statistical power and strengthening the results of the reviewed studies and enhancing the chance of recognizing a true effect of exposure [85].

## Conclusions

To the best of our knowledge, this is the first meta-analysis evaluating oral health in PWUD and the present study provides important data regarding significantly higher levels of dental health problems among PWUD. Screening for oral health disease in drug treatment settings could increase early detection of oral health problems and facilitate referral to dental care services. Our study identified poor DMFT and caries and periodontal diseases among PWUD which may be explained by irregular tooth brushing and a long history of drug use. There is evidence to show that it is possible to treat caries and periodontal complications among PWUD by linking substance treatment programs with oral hygiene services. Addressing dental health issues among PWUD is vital, and despite o the analytical challenges of segregating the direct impact of drug use on oral health morbidities. The data highlight the need for developing affordable and accessible prevention programs that improve the oral health status among PWUD by the policymakers and public health authorities.

## Data Availability

The datasets used and/or analyzed during the current study are available from the corresponding author on reasonable request.

## Abbreviations

BOP: Bleeding on probing
CI: Confidence interval
DMFT: Decayed, missing and filled teeth
JBI: The Joanna Briggs Institute
MA: Methamphetamine
MT: Missing teeth
NHANES: National Health and Nutrition Examination Survey
OR: Odds Ratio
PRISMA: Preferred for Systematic Reviews and Meta-Analyses
PSI: Periodontal screening index
PWDNUD: People who do not use drugs
PWUD: People who use drugs
TD: Tooth decay
